# USP32 promotes tumorigenesis and chemoresistance in gastric carcinoma via upregulation of SMAD2

**DOI:** 10.7150/ijbs.43117

**Published:** 2020-03-12

**Authors:** Ning Dou, Qingqing Hu, Li Li, Qiong Wu, Yandong Li, Yong Gao

**Affiliations:** Department of Oncology, Shanghai East Hospital, Tongji University School of Medicine, Shanghai 200120, China.

**Keywords:** USP32, SMAD2, gastric carcinoma, drug resistance

## Abstract

USP32, a member of the ubiquitin-specific proteases family, has been implicated in the development of breast cancer and small lung cancer. However, its biological functions and clinical significance in gastric cancer (GC) remain unclear. In the present study, we reported that knockdown or depletion of USP32 significantly inhibited GC cell proliferation and migration *in vitro* and *in vivo,* indicating that USP32 functions as an oncogene in GC. Importantly, results from immunohistochemical staining in a tissue microarray revealed that USP32 was upregulated in GC tissues compared with paracancerous tissues. Further analyses showed that high expression of USP32 was closely related with high T-staging and poor outcomes of GC patients. Mechanistically, USP32 silencing caused a decrease in the expression of SMAD2, which resulted in the inhibitory effects of GC cells on growth, motility, and chemoresistance to cisplatin. Therefore, our findings strongly suggest the involvement of USP32 in GC progression and provide a potential target for future therapy of GC.

## Introduction

Protein ubiquitylation, one of the post-translational modifications (PTMs), plays important role in regulating protein degradation, signal transduction, and DNA damage responses 1,2. Ubiquitylation is a reversible process, and generally ubiquitin was added to substrates by the E1 activating enzymes, E2 conjugating enzymes and E3 ligating enzymes then removed by deubiquitylating enzymes (DUBs) 3. Deubiquitylating enzymes (DUBs) have been recognized as powerful tools for altering stability, localization, and activity of proteins, which are widely implicated in pathological mechanisms of many human diseases 4-6.

Nearly 100 DUBs are identified in the mammalian genome, and there are several subclasses of DUBs. Structurally, ubiquitin carboxy (C)-terminal hydrolases (UCHs), ubiquitin-specific proteases (USPs), Machado-Joseph disease protein domain proteases (MJDs) and ovarian tumor proteases (OTUs) are papain-like cysteine proteases, while JAB/MPN/Mov34 metalloenzyme (JAMM) domain proteases are zinc-dependent metalloproteases 7. The USP subfamily, with more than 50 members in humans, has been reported to play pivotal roles in tumors 8-10. USP28 is identified as a key regulator of cell proliferation in colorectal cancer 11. USP22 can function as a tumor driver gene via regulating cell cycle 12. USP46 is selectively recruited by the high-risk HPV and promotes cell proliferation in high-risk HPV caused cervical cancer 13. On the contrary, USPs can also be recognized as tumor suppressors, such as USP10, USP17 and USP49 have been implicated in several cancer types 14-16.

USP32, a new member of the USP subfamily, is an ancient and highly conserved gene located on 17q23. It is reported to be the origin of Tre2 (USP6) oncogene, and nucleotides 3194-6063 of USP6 mRNA have 97% sequence identity to USP32 17. Some reports have revealed its involvements in cancer. In fact, USP32 exhibits overexpressed in breast cancer and human small cell lung cancer and may serve as an oncogene through promoting cell proliferation and tumor metastasis 18,19. However, little is known about the function and expression of USP32 in gastric cancer.

Emerging studies have indicated key roles for ubiquitin modification in transforming growth factor-β (TGF-β) signaling 20. For example, the E3 ligase TRIM33/Ectodermin has been shown to inhibit ubiquitination of SMAD4 21. In addition, Smurf2 binds to Smad7 forms a SMAD7-SMURF2 complex which activates and degrades TGF-β receptor 22. In this study, we partially set up a link between USP32 and SMAD2, a key component of TGFβ signaling pathway, in GC development and progression. We found that USP32 affected the SMAD2 expression partially in an ubiquitin protease dependent manner. Functionally, SMAD2 may mediate the oncogenic effects of USP32 on cell growth, metastasis and drug resistance. Moreover, we also found that USP32 is upregulated in gastric cancer tissues and its expression has strong clinical relevance. Taken together, these results provide a novel insight of USP32/SMAD2 axis in GC progression.

## Materials and methods

### Cell culture

GC cell lines, MGC803 and SGC7901 were purchased from the cell bank of the Chinese Academy of Sciences at Shanghai, China. GC cells were grown in Dulbecco's Modified Eagle's medium (DMEM, Corning, US) containing 10% fetal bovine serum (FBS) supplemented with penicillin/streptomycin (100µg/mL). The cells were maintained at 37°C and 5% CO_2_.

### Colony formation assay

GC cells (2,000 cells per well) were plated in 6 well plates and incubated for 14-20 days in triplicate. Then colonies were washed with PBS and stained with 0.5% crystal violet for 15 min. The visible colonies were photographed and counted results were obtained from three independent experiments.

### Cell proliferation assay

For proliferation assay, GC cells (3,000 cells per well) were seeded into 96-well culture plates in triplicate and incubated for 5 days at 37°C in a humidified incubator with 5% CO_2_. Every 24 h interval, 10 µl of Cell Counting kit-8 (CCK-8; Dojindo Laboratories, Japan) was added to each well and incubated at 37°C for 1 h. Then the plates were read at 450 nm (SpectraMax M5, Molecular Devices, US). All these experiments were repeated at least three times.

### Transwell chamber assay

For *in vitro* migration assay, cells (30,000 cells per well) were planted in the upper chambers (Corning, US) in serum free medium in 24-well plates. The bottom was added with DMEM plus 10% FBS (fetal bovine serum). After cultured 24-36 h, cells on the undersurface were stained with crystal violet and calculated under microscopy. Each experimental group included three replicates.

### Western blotting

Cells were lysed in RIPA buffer, and protein concentration was measured using a BCA Assay Kit (Thermo Fisher Scientific, US). 20μg proteins were electrophoresed by SDS-polyacrylamide gels and transferred onto a nitrocellulose membrane (Millipore, US). The membrane was then blocked in non-fat milk for 1 h at room temperature and subsequently incubated with the primary antibodies overnight at 4°C. After washing with PBST (phosphate-buffered saline containing 0.05% Tween 20) for three or four times, then the membrane was incubated with the secondary antibody for 1 h at room temperature. Protein bands were visualized using the Odyssey Infrared Imaging System (Li-COR Biosciences). The antibodies used in this study were as follows: USP32 (sc-374465, Santa Cruz Biotechnology, 1:100), β-actin (#81178, Santa Cruz Biotechnology, 1:1,000), SMAD2 (#12570-1-AP, Proteintech, Wuhan, China, 1:500), p-SMAD2 (#18338, Cell Signaling Technology, US), and FLAG (F1804, Sigma-Aldrich, US).

### RNA interference and SMAD2 construct

GC cells were transfected with siRNAs or plasmids using Lipofectamine 3000 (Invitrogen; Thermo Fisher Scientific) in accordance with the manufacturer's instructions. All of siRNAs used in this study were purchased from Gene Pharma, Shanghai, China. The siRNAs were designed as follows: 5′-UUCUCCGAACGUGUCACGUdTdT -3' (si-NC, as a negative control) 5'-GACCUGUGGACUCUCAUAUTT-3' (si-USP32-homo-386); 5′-GGACAGUUAUAUGCACUUATT-3' (si-USP32-homo-3029). Lentiviral particles for knockdown of USP32 were packaged from GenePharma, Shanghai using above corresponding sequences (si-USP32-homo-386 and siNC). The SMAD2 expression plasmid fused a C-terminal FLAG was purchased from ViGene Bioscience, China (#CH809987).

### Cas9 construction

The Lentiviral CRISPR/Cas9 system sgRNAs were synthesized and sub-cloned by Shanghai GenePharma, China. The sgRNA sequences targeting USP32 was 5′-GGTTATTGAAGGCTCAATCCCGG-3′ (sgUSP32) and a negative control 5′-TCGTACTCTACAGCAGATGC-3′ (sgNC). The MGC803 and SGC7901 cells were seeded in 6-well plates and infected with the Cas9 lentivirus, respectively. After puromycin selection, MGC803-sgUSP32, MGC803-sgNC, SGC7901-sgUSP32 and SGC7901-sgNC were generated.

### Quantitative real-time PCR (qRT-PCR)

Total RNAs from cells or tissues were extracted using TRIzol reagent (Sigma-Aldrich) and reverse transcription was conducted by the Prime script™ RT Reagent kit with gDNA Eraser (Takara, Japan). The expressions of the target genes were evaluated by qRT-PCR on Applied Biosystems 7500 Fast Real-Time PCR system with Power TB Green PCR Master Mix (TaKaRa, Japan). The following primers were used for qRT-PCR assays: β-actin-qF, 5-CCTGGCACCCAGCACAATG-3 and β-actin-qR, 5-GGGCCGGACTCGTCATACT-3; SMAD2-qF, 5-ATGTCGTCCATCTTGCCATTC-3 and SMAD2-qR, 5-AACCGTCCTGTTTTCTTTAGCTT-3.

### Patients' specimens

The tissue microarray that contains 314 GC tumor specimens and 22 non-tumor specimens was purchased from Alenabio Company (MC6162, Xi'an, China). These tissue samples included the pathologic grade and the clinical stage. USP32 expression was assessed by immunohistochemical staining following the standard immunohistochemical procedures and the staining results were double-blindly scored. A total of 36 pairs of gastric cancer samples were gathered at Shanghai East Hospital from 2013 to 2015 with informed consents. All the samples were snap-frozen in liquid nitrogen after resection and stored at -80°C prior to RNA extraction. The study for use of clinical samples was approved by the Medical Ethics Committees of Shanghai East Hospital.

### Animal experiments

Male nude mice (4-5 weeks, BALB/c) were purchased from SLAC Laboratory Animal Center at Shanghai, China. All animals received standard care, and protocols for study were approved by the Medical Ethics Committee of Shanghai East Hospital. For xenograft model of gastric cancer, 2×10^6^ stably infected cells were injected subcutaneously and bilaterally into the flank of nude mice (n=8). After 21 days, the tumors were isolated and weighed. For tumor metastasis assay, the tail vein of each mouse was injected with 1.5×10^6^ cells (n=5). After 28 days, mice were euthanized and the tumor nodules on the lung surface were calculated. Hematoxylin and eosin (H&E) of lung tissues was performed and photographed.

### Path scan intracellular signaling array

A primary PathScan® Intracellular Signaling Array Kit #12923 (Cell Signaling Technology, US) was employed to detect the intracellular signaling according to the manufacturer's instructions. The result was visualized using the Odyssey Infrared Imaging System and quantified by Image Studio software.

### Luciferase assay

For reporter assays, USP32 silenced cells and negative control cells were plated in 24-well plates and transfected with 2 ug SMAD-Luc reporter plasmid and 50 ng of pRL-SV40 Renilla luciferase construct. 24 h after transfection, SMAD Response Element (SRE) activity was measured using a dual luciferase assay kit (Promega, USA).

### Flow cytometry analysis

The Annexin V-FITC/PI apoptosis detection kit (KGA-107, KeyGEN, China) was used to detect the cell apoptosis following the manufacturer's instructions. Data was analyzed by flow cytometer and the experiment was repeated three times.

### Statistical analysis

Quantitative data are presented as the mean± S.E.M. or mean± S.D., statistical significance was determined by Student's t-test or *X*^2^ test. Statistical significance is represented in figures by: * *P* < 0.05; ** *P* < 0.01.

## Results

### Silencing of USP32 inhibits GC cell growth and migration* in vitro*

**Figure 1:**
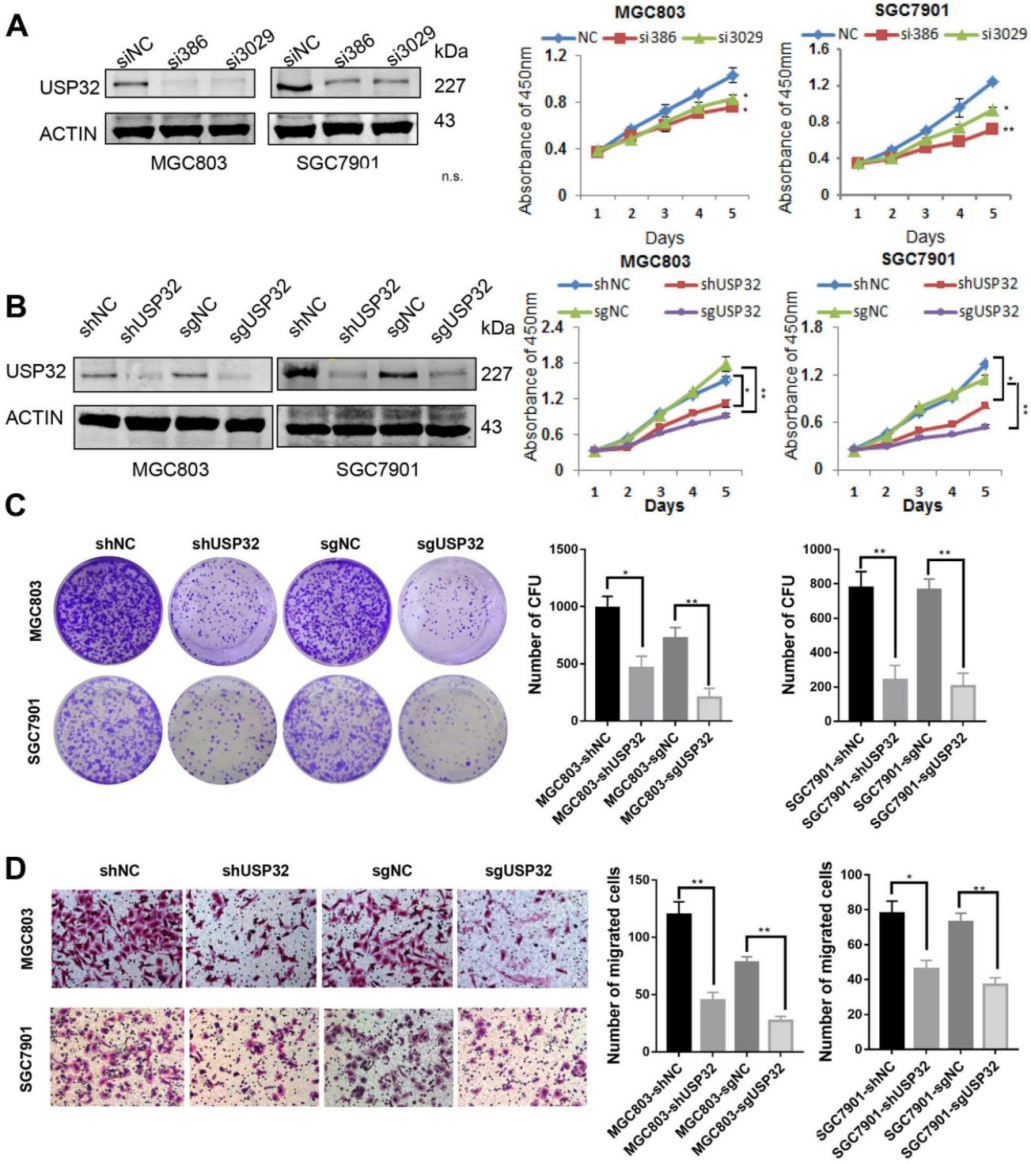
** Knockdown of USP32 inhibited GC cell growth and migration *in vitro.* (A)** The knockdown efficiency of USP32 was determined by western blotting (siUSP32-386 and siUSP32-3029). CCK8 assays were performed in the cells as indicated.** (B)** Western blot and CCk8 assays were performed in stable cell lines infected with lentivirus (LV-shUSP32 or control LV-shNC; LV-sgUSP32 or control LV-sgNC). **(C)** Cell colonies were stained and counted two weeks after plating GC cells as indicated into 6-well plates. **(D)** Knockdown or depletion of USP32 decreased the number of migrated cells in MGC803 and SGC7901 cells. *P< 0.05, **P< 0.01.

USP32 expression was knocked down in MGC803 and SGC7901 cells by transient transfection with siRNAs against USP32, and result of western blot analysis indicated that siUSP32-386 and siUSP32-3029 markedly decreased USP32 expression (Figure 1A). Subsequently, CCK-8 assay was employed to investigate the effect of USP32 knockdown on cell growth. The results demonstrated that transient silencing of USP32 in MGC803 and SGC7901 cells significantly reduced cell growth rate (Figure 1A). To further confirm the function of USP32 in GC cells, stable cell lines were established by two approaches. One was lentivirus-mediated USP32 knockdown with shUSP32-386, the other was lentivirus-mediated depletion of USP32 based on CRISPR/Cas9 technique. As shown in Figure 1B, growth curve results revealed that stable silencing or depletion of USP32 inhibited the proliferation capacity of GC cells. Consistently, downregulation of USP32 also led to an inhibitory effect on colony formation of those stable cells (Figure 1C). In addition, we observed that cell migration ability was decreased in USP32 downregulated GC cells by a Transwell chamber analysis (Figure 1D). These collective data suggested the critical roles of USP32 in promoting cell proliferation and migration* in vitro*.

### Silencing of USP32 attenuates tumor growth and metastasis of GC cells *in vivo*

**Figure 2:**
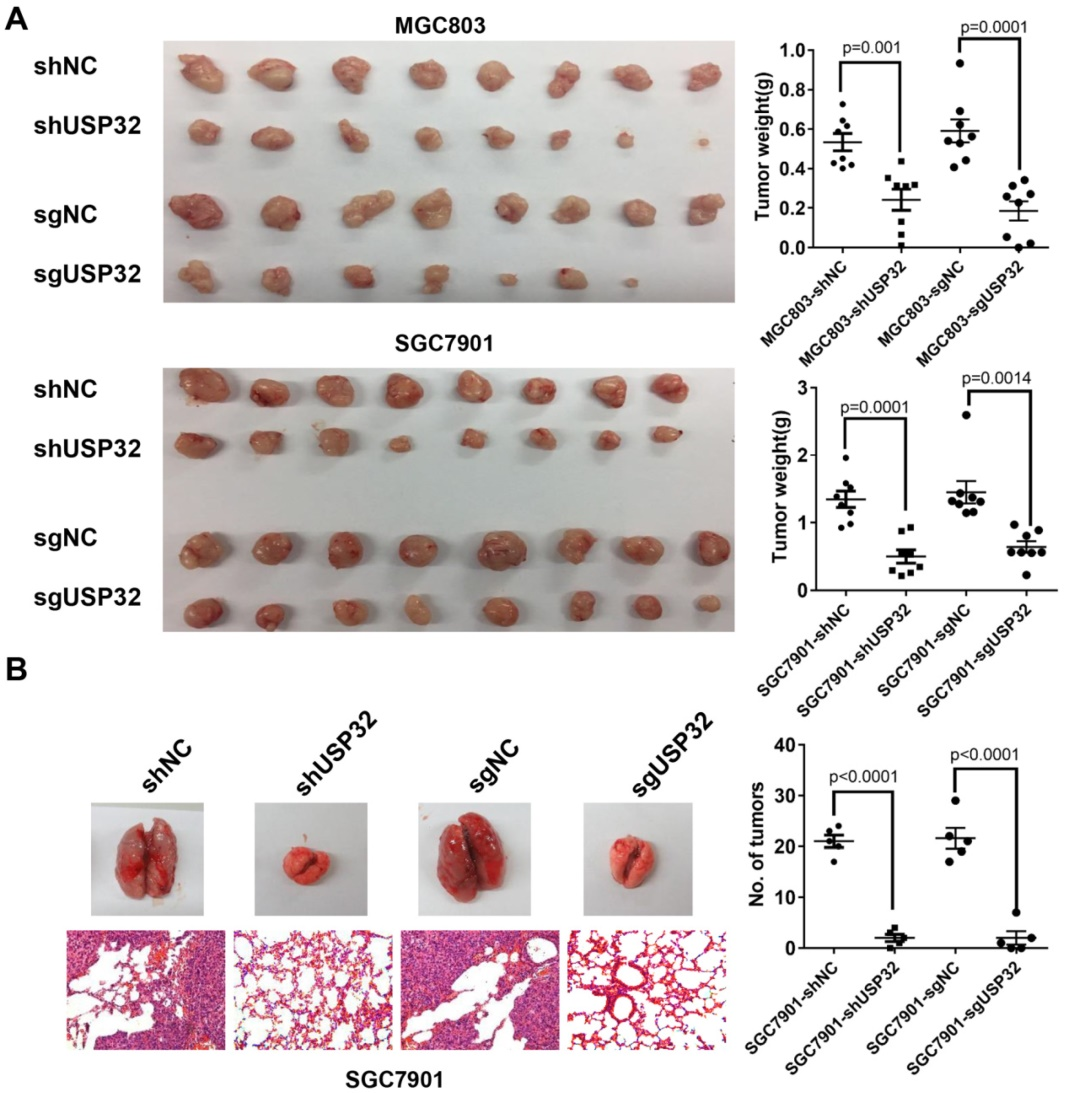
** Knockdown of USP32 suppressed the tumorigenicity and metastasis of GC cells *in vivo.* (A)** Knockdown of USP32 decreased the tumorigenicity of MGC803 and SGC7901 cells in nude mice. MGC803 and SGC7901 cells stably expressing shUSP32 or sgUSP32 were subcutaneously injected into nude mice. Mice were sacrificed after 3 weeks and tumor weights were measured. Values are expressed as mean ± SD (n=8). **(B)** Knockdown of USP32 inhibited lung metastasis of SGC7901 cells. Lung metastatic tumor nodules on lung surface were counted and lung sections were hematoxylin eosin (HE) stained (n=5, Magnification: ×200).

To further address the effect of USP32 on tumor growth *in vivo*, a tumor xenograft model was established and cells with stable downregulation of USP32 and corresponding control cells were subcutaneously injected into 4-week-old male nude mice (n=8 per group), and tumor tissue were harvested 3 weeks after injection. As expected, the tumors in USP32 downregulation group exhibited lower mass and smaller size than those in control group (Figure 2A), suggesting that USP32 is required for tumorigenicity of GC cells *in vivo*. Moreover, pulmonary metastasis assays were performed and the results showed that downregulation of USP32 markedly decreased metastatic foci on lung surfaces (Figure 2B). These data further confirmed the oncogenic role of USP32 in animal models.

### USP32 is upregulated in gastric cancer tissues and is related to tumor stage

**Figure 3:**
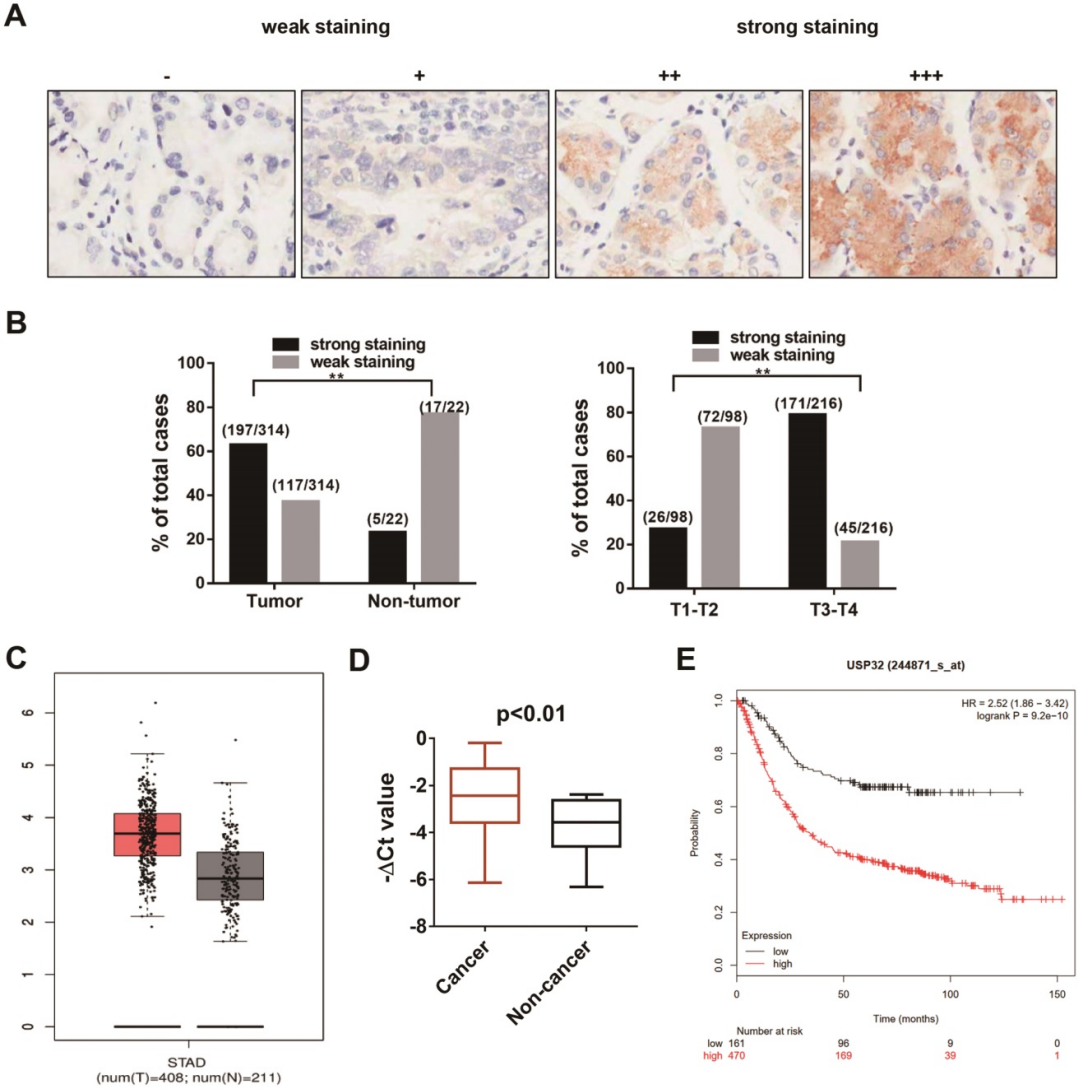
** The expression pattern of USP32 in gastric cancer tissues. (A)** The expression of USP32 was determined by immunohistochemical staining on sections of gastric cancer tissues. “-” (negative); “+” (slightly positive) “++” (moderately positive); “+++” (strongly positive). Magnification: ×200. **(B)** The ratio for strong stating (++, +++) and for weak staining (-, +) in tumor and non-tumor tissues (left) and in T1-T2 stage and T3-T4 stage (right). **P < 0.01. **(C)** The expression of USP32 from TCGA database. **(D)** qRT-PCR was conducted to explore the expression of USP32 in 36 pairs of gastric cancer samples. **(E)** Kaplan-Meier plot displayed the overall survival patients with high (red line; n=470) and low (black line; n=96; P<0.01, Log-rank test) expression of USP32.

To explore the expression pattern of USP32 in GC, we performed an immunohistochemical (IHC) analysis using USP32 specific antibody in a human gastric cancer tissue microarray, which contains 314 tumor samples and 22 non-tumor tissues. As shown in Figure 3A, the expression level of USP32 was graded by intensity of IHC staining. The strong staining of USP32 was far more frequently observed in gastric cancer tissues (197/314, 62.7%) than in para-carcinoma normal tissues (5/22, 22.7%). Furthermore, there was a significant correlation between USP32 expression and T staging (Figure 3B, *P*<0.001). Consistent with our IHC results, high expression of USP32 was found in tumor tissues by analyzing TCGA database using GEPIA online tools (http://gepia.cancer-pku.cn/) (Figure 3C) and performing qRT-PCR assay in 36 pairs of gastric cancer samples (Figure 3D). Additionally, by analyzing Kaplan-Meier Plotter datasets (http://kmplot.com/analysis/index.php?p=service&cancer=gastric), USP32 expression was shown to be significantly correlated with poor prognosis of gastric cancer patients (Figure 3E). These results strongly suggested that USP32 is frequently overexpressed in GC and could act as a biomarker for GC.

### USP32 regulates SMAD2 expression in GC cells

**Figure 4:**
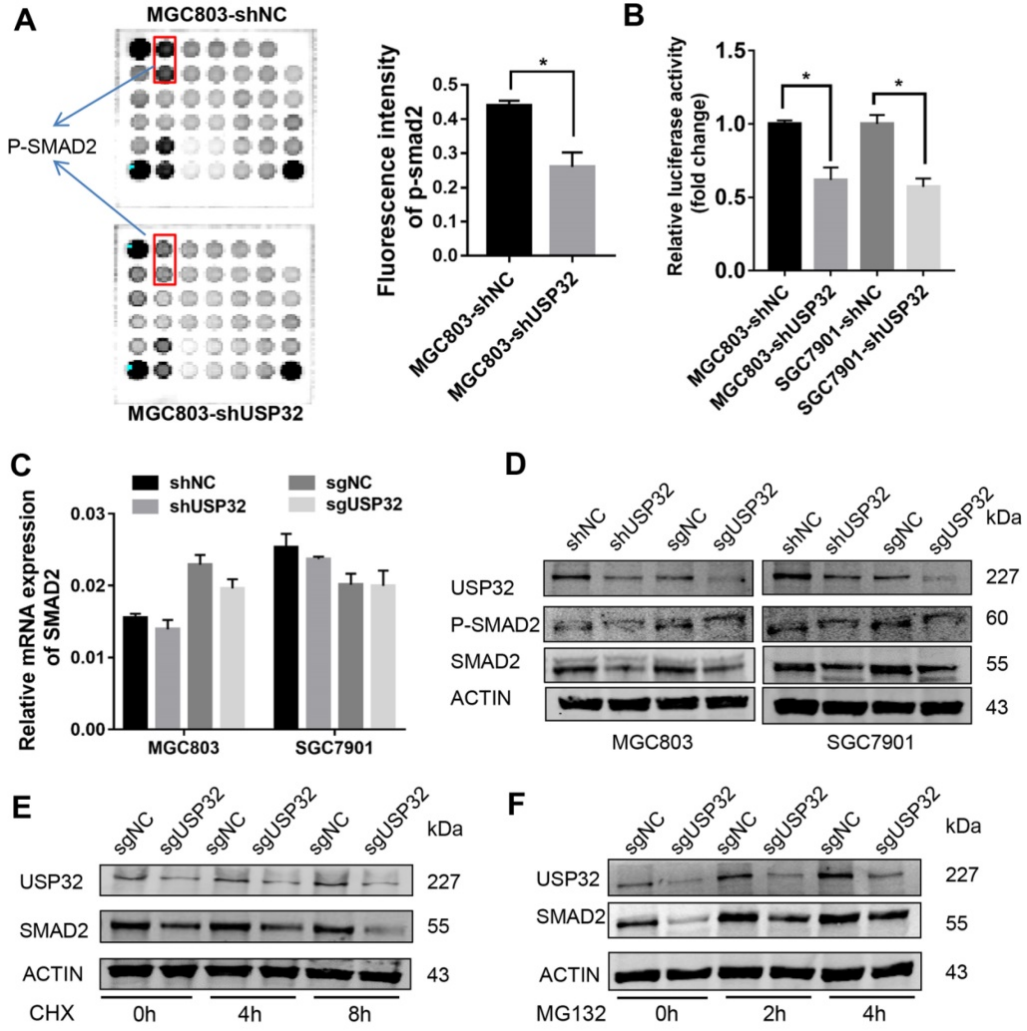
** Effects of USP32 downregulation on the expression of SMAD2 in GC cells. (A)** Intracellular signaling array was performed in MGC803 cells. The p-SMAD2 dots was marked with rectangles and quantified by Image Studio Version 3.1. **(B)** Relative luciferase activity of MGC803 or SGC7901 cells in which USP32 was stably downregulated. SMAD2/3 response element (SRE) luciferase reporter and a Renilla luciferase plasmid were co-transfected. *P < 0.05.** (C)** The mRNA level of SMAD2 was detected by qRT-PCR in MGC803 and SGC7901 cells. The data were normalized with the β-actin mRNA. **(D)** Western blot analysis was used to confirm the p-SMAD2 and SMAD2 protein level in MGC803 and SGC7901 cells as indicated. **(E)** MGC803 cells stably expressing control (sgNC) or USP32 sgRNAs were treated with CHX, and collected at the indicated times, then western blot analysis was performed. **(F)** MGC803 cells stably expressing control (sgNC) or USP32 sgRNAs were treated with MG132, and collected at the indicated times, then western blot analysis was performed.

A PathScan® intracellular signaling array was employed to elucidate the downstream effectors of USP32 in GC cells. As shown in Figure 4A, phosphorylated (p-) SMAD2 level was significantly reduced in USP32 silenced MGC803 cells. To validate this result, dual luciferase reporter assays were performed with SMAD2/3 response element reporter plasmid, and the results indicated that the luciferase activity was markedly decreased with the absence of USP32 (Figure 4B). qRT-PCR results demonstrated that USP32 downregulation had no effect on SMAD2 transcription (Figure 4C). Interestingly, when we detected the expression level of p-SMAD2 via western blot analysis, we found that the level of p-SMAD2 was decreased accompanied by a reduction of total protein level of SMAD2 in USP32 downregulated cells (Figure 4D). Accordingly, GC cells were treated with cycloheximide (CHX), a reagent for blocking *de novo* protein synthesis. Results from western blots indicated that downregulation of USP32 dramatically decreased the SMAD2 protein level after protein synthesis was inhibited (Figure 4E). As the same time, the decrease in SMAD2 protein level induced by USP32 downregulation could be rescued by the proteasome inhibitor MG132 (Figure 4F). These results suggested that USP32 may regulate SMAD2 expression by enhancing protein stabilization of SMAD2.

### SMAD2 restores USP32 knockdown induced growth and migration defects

**Figure 5:**
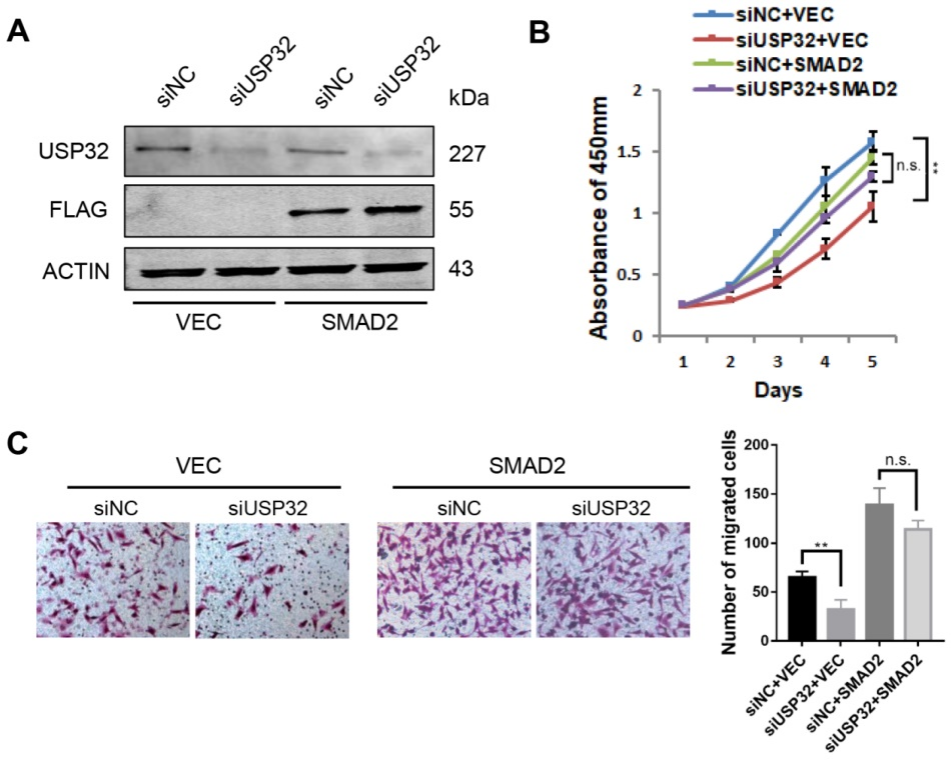
** SMAD2 rescued phenotypic defects in cell growth and migration caused by downregulation of USP32. (A)** Western blot analysis was performed to confirm the overexpression of SMAD2 and the silencing of USP32. **(B)** Silencing of USP32 significantly inhibited cell growth in MGC803 cells while overexpressed SMAD2 attenuated the inhibitory effect. **(C)** Transwell assays were used to detect the effects of USP32 and SMAD2 expression on cell migratory ability in MGC803 cells.

To investigate the functional relevance of USP32 and SMAD2, we overexpressed SMAD2 together with knockdown of USP32 in MGC803 cells (Figure 5A). The CCK8 assays revealed that overexpression of SMAD2 significantly attenuated the inhibitory effect on cell proliferation caused by knockdown of USP32 (Figure 5B). Similarly, Transwell chamber assays also proved that knockdown of USP32 decreased the number of migrated cells while overexpression of SMAD2 compensated this phenotype (Figure 5C). Therefore, these data indicated that SMAD2 could restore the oncogenic effects of USP32 in GC cell proliferation and migration.

### USP32 confers drug resistance of GC cells

**Figure 6:**
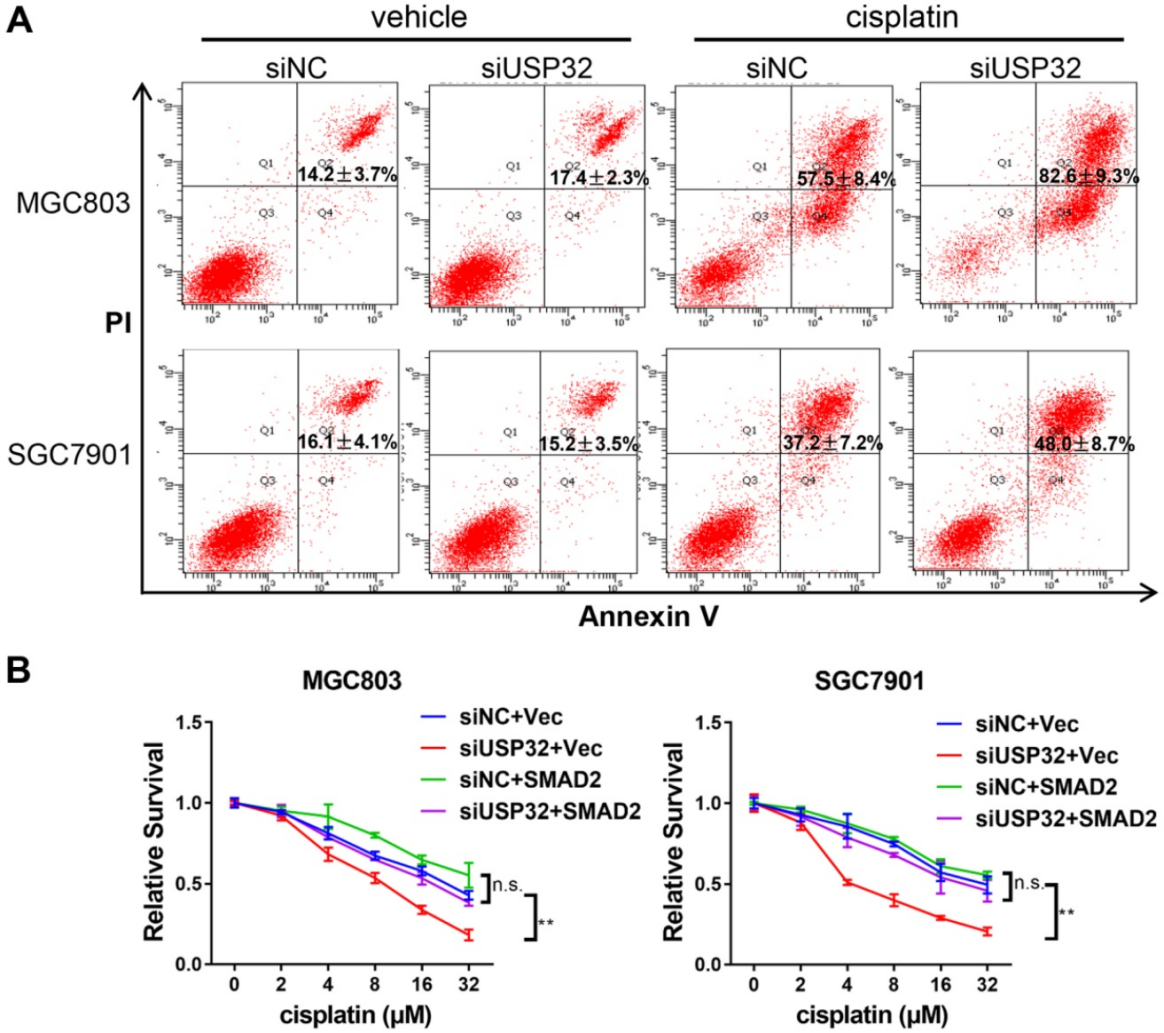
** USP32 downregulation sensitized GC cells to cisplatin*.* (A)** 48 h after cisplatin treatments, flow cytometry analysis was performed in MGC803 and SGC7901 cells transfected with si-NC or si-USP32. **(B)** Cells with USP32 knockdown or SMAD2 overexpression were treated with cisplatin as indicated concentrations for 48 h, and then cell viabilities were determined by CCK8 assays.

It has been reported that TGFβ/SMAD signaling pathway involved in cisplatin resistance of tumor cells 23,24. Considering USP32 regulates SMAD2 expression, we attempted to elucidate whether USP32 could mediate this process. By flow cytometric analyses, we demonstrated that cisplatin-induced cell death was increased in USP32 knockdown cells compared with control cells (Figure 6A). Furthermore, knockdown of USP32 attenuated the resistance of GC cells to cisplatin in CCK8 assays, whereas overexpression of SMAD2 partially rescued the defect of USP32 silenced GC cells in cisplatin resistance (Figure 6B), suggesting that USP32 may confer chemoresistance in GC.

## Discussion

Gastric cancer still remains the third leading cause of cancer-related death despite the advances that have been made in diagnosis and treatment 25. Due to the limited therapeutic options, late diagnosis and uncontrolled recurrence, the 5-year survival rate for GC patients is very low. Therefore, it is urgent to develop new and more effective therapeutic targets for GC. In the present study, we identified USP32, a new ubiquitin-specific protease, as an oncogene involved in GC cell growth and metastasis.

Our data firstly demonstrate that USP32 downregulation significantly inhibited GC cell proliferation and migration *in vitro*, as well as in a mouse model. These results are consistent with previous report in breast cancer and small lung cancer 18,19, suggesting that USP32 may function as an oncogene in tumors. Meanwhile, we also show that USP32 was upregulated in GC tissues and high expression of USP32 was related to low overall survival of GC patients and high T staging. The findings raise the possibility that USP32 might serve as a potential biomarker and therapeutic target for GC.

Next, we provide evidence that knockdown of USP32 decreased the expression of SMAD2. SMAD2, a vital mediator of TGF-β signaling pathway, is subjected to regulation in changing subcellular localization and transcriptional response 26. SMAD2 has been reported to be abnormal expression in many cancers. Tang et al reported that profilin-2 (Pfn2) enhances Smad2/Smad3 expression, and high SMAD expression correlates with poor outcome of lung cancer patients 27. In addition, several investigations have indicated the oncogenic roles of SMAD2 in pancreatic cancer, gastric cancer, and prostate cancer 28-30. However, it is controversial for the functional roles of SMAD2 in cancer. In other reports, SMAD2 was shown to exert tumor suppressor function during tumorigenesis 31,32. To date, the exact functions and regulatory mechanisms of SMAD2 in cancer are complicated and not fully understood. In our case, we found the protein expression of SMAD2 is no longer affected by USP32 after treatment with proteasome inhibitor MG132. This suggests that SMAD2 is regulated by USP32 through proteasome pathway, although we did not find any direct interaction between SMAD2 and USP32 via co-immunoprecipitation experiments (Data not shown). The rescue experiments also indicate that SMAD2 severs as the downstream of USP32 and could restore at least partial defects in growth and migration of USP32 silenced cells.

Many researches have shown deubiquitylating enzymes participate in chemoresistance 33,34. Song et al reported that deubiquitylating enzyme Rpn11 can enhance the chemosensitivity of bortezomib in multiple myeloma cells 35. Other than that, Qin et al demonstrated that silencing of USP37 can reduce resistance to cisplatin-targeting therapies in breast cancer 36. In this regard, we also observed that USP32 downregulation could decrease the cisplatin-resistance in GC cells, suggesting the involvement of USP32 in drug resistance. In the meanwhile, overexpression of SMAD2 can enhance the resistance to cisplatin in USP32 silenced cells. These observations further support the previous proposal that SMAD2 functions as downstream of USP32 in GC.

In conclusion, our findings provide a new insight into the expression and function of USP32 in cancer. SMAD2 may mediate USP32-induced cell growth, metastasis and chemoresistance. Targeting USP32 might be a potential therapeutic strategy for GC.
